# Factor structure and psychometric properties of an adapted HIV stigma tool for measuring disability-related stigma among smallholder farmers in Western Kenya – Findings from a cross-sectional study

**DOI:** 10.1371/journal.pone.0345597

**Published:** 2026-03-26

**Authors:** Anita Jeyam, Elena Schmidt, Stevens Bechange, Stephen Pye, George Okello, Sheru Muuo, Moses Chege, Emma Jolley

**Affiliations:** 1 Sightsavers United Kingdom, Haywards Heath, United Kingdom; 2 Sightsavers Kenya Country Office, Nairobi, Kenya; Universiti Sains Malaysia - Kampus Kesihatan, MALAYSIA

## Abstract

**Background:**

People with disabilities face stigma and discrimination which contribute to barriers and poor outcomes in many areas such as health or employment. There is currently a dearth of tools for measuring disability-related stigma and discrimination in low-and middle-income countries.

**Aims:**

This study aims to evaluate the construct validity and internal consistency of adapted human immunodeficiency virus (HIV) stigma and discrimination tools for use in the context of disability, among smallholder farmers in Western Kenya.

**Methods:**

Separate tools were used to measure enacted stigma in the community among people without disabilities, and stigma experienced by people with disabilities. We split the relevant sample in two (N = 3642 for enacted stigma; N = 710 for experienced stigma), using exploratory factor analysis (EFA) on the first half and confirmatory factor analysis (CFA) on the second.

**Results:**

For enacted stigma, a two factor-structure was identified: negative attitudes towards people with disabilities and positive/supportive attitudes towards people with disabilities. For experienced stigma, the scale was reduced to six items capturing interpersonal stigma, and was unidimensional. CFA model-fit indices were good. McDonald’s omega coefficient indicated good internal consistency for the enacted stigma scale (0.79) and for the experienced stigma scale (0.82).

**Conclusion:**

The adapted HIV stigma scale showed good properties in terms of construct validity and reliability, for measuring aspects of disability-related stigma. These are promising results to build on to develop locally valid and standardised disability-related stigma and discrimination measurement tools.

## Introduction

Disability-related stigma has been identified widely as one of the major causes of discrimination and exclusion of the estimated 1.3 billion people living with disabilities globally [1, 2]. People with disabilities have been observed to have worse educational, economic, and health outcomes, than people without disabilities [1, 3, 4]. While much of that inequality may be attributed to inaccessible environments that fail to accommodate individuals’ needs, it combines with, and indeed is driven by, stigma, a socially conferred judgement that some persons or groups are tainted and “less than” [5–7].

A recent systematic review of disability-related stigma and discrimination in sub-Saharan Africa and South Asia [8] highlighted the extent to which stigma plays a huge role in barriers preventing the equitable participation of people with disabilities in all areas of life, for example hindering them from accessing services, isolating and marginalizing and trapping them in poverty. The fear of experiencing stigma and discrimination can in turn lead to extreme isolation of people with disabilities [9]. Stigma has also been reported as a barrier to the implementation of the convention on the rights of persons with disabilities in Africa [10]. Traditional cultural beliefs where disability is associated with witchcraft or malevolent attacks from spirits for example can reinforce social distancing and discrimination [10, 11], noting however that traditional beliefs can also be positive. For example, some communities in Turkana, Kenya view a child with disability as a gift from God who should be well cared for [10].

The systematic review [8] highlighted major methodological limitations in disability stigma research including a lack of conceptual clarity around stigma, a lack of data on the magnitude and impact of stigma, and a lack of tools for measuring different aspects of stigma in a valid and reliable way. The latter is a particular challenge to addressing stigma, as without appropriate measurement tools, stigma cannot be clearly identified and enumerated, interventions to reduce it cannot be evaluated, and findings across studies cannot be compared in a straightforward manner.

With the increasing focus of international development programmes on measuring equity and disability inclusion, there has been a strong drive to use consistent measurements of stigma to better understand the prevalence and distribution of stigma in different social contexts and in people with different types of impairments and to evaluate how it changes over time [1, 12].

Historically, quantitative studies of stigma used scales developed for specific diseases, for example leprosy, tuberculosis, mental health disorders or human immunodeficiency virus (HIV)/acquired immunodeficiency syndrome (AIDS). Very few studies have tried to apply tools across different types of health conditions or develop a universal tool applicable to all settings [13, 14]. Attitudes towards disability have been reported to vary widely across countries and communities, as well as social contexts. In addition to this, stigma itself varies across types of, severity and origin of disability, with some disabilities considered more acceptable than others, often depending on their visibility and impact on functioning [15–17]. Consequently, when a tool developed for one context and one health condition or impairment is applied to another context or condition(s), it is important to collect validity evidence and understand how the tool performs in these specific circumstances [18].

In this paper, we report results of factor analyses performed on two adapted stigma measurement instruments (scales) used to assess enacted and experienced stigma in the community of sorghum farmers in Western Kenya. Enacted stigma refers to negative attitudes and behaviours towards people with disabilities reported by the general population (people without disabilities), while experienced stigma refers to negative attitudes and behaviours reported as experienced by people with disabilities themselves. The scales were adapted from existing stigma scales developed by Nyblade, MacQuarrie et al. [19] and Stangl, Lilleston et al. [20] for assessing HIV/AIDS-related stigma in settings that may be comparable to Western Kenya.

While HIV-related stigma scales have been applied and validated in various settings, there is a dearth of data on disability-related stigma scales, particularly in low-income rural settings. To the best of our knowledge, this is the first paper to examine the properties of adapted HIV stigma scales in a disability context among smallholder farmers in Kenya.

## Materials and methods

### Study design and setting

The stigma module was a secondary component of a broader baseline survey from a non-randomised cluster field trial. This trial primarily aimed to evaluate the impact of a five-year inclusive farming development programme on labour outcomes among smallholder farmers in Western Kenya.

Participants were recruited in the community using a two-staged sampling with sampling clusters selected with probability proportionate to size and households by systematic sampling. The study population comprised adults aged 18 and older residing in the study communities, who had access to farmland. Within the households, all eligible adults were offered participation. Although this study design originally allowed for recruiting more than one individual per household, in practice, most households had only a small area of land dedicated to sorghum. As a result, only one person per household met the inclusion criteria. This sampling strategy was chosen to achieve a representative sample of the communities studied and to minimise selection bias. Study design, setting and sampling process have been previously described by Bechange et al. [21].

Disability status was ascertained using the Washington Group-International Labor Organization (ILO) Labor Force Survey Disability Module (LFS-DM), based on the levels of self-reported difficulties in functioning [22]. An individual was considered with disability if they responded “cannot do at all” or “a lot of difficulty” to any of the questions on seeing, hearing, walking, concentrating, self-care or communicating, or “daily” to any of the anxiety or depression questions. We chose to adopt this definition of disability based on the recommendation of the Washington Group on Disability Statistics, to reflect the multidimensional nature of disability and the fact that both functional and psychosocial difficulties can restrict participation in society and, in particular, affect employment outcomes, which were one of the main focus of the overarching project this study was part of.

In total there were 4,459 participants in the sample. Data on experienced stigma was collected from 767 participants with disability. Data on enacted stigma was collected from 3,682 participants without disability.

Data were collected over a 7-week period, recruitment into the study took place from 26^th^ January to 18^th^ March 2022.

### Stigma measurement tools

We prioritised and modified questions from the HIV/AIDS related stigma scales [19, 20] to ensure relevance to disability as a stigmatising condition and ease of comprehension across the three main languages spoken in the study area: Swahili, Luo, and Luhya. We also asked some questions about experiences of support because we wanted to minimise the potential for extreme response and social desirability bias, given the high exposure to HIV-related research utilising scale items in this setting [23–25]. We recognise that these positive experiences are conceptually ambiguous as they are reflective of social support. Social support given to people with disabilities can be influenced by stigma. We therefore included these positive/supportive items in our scale for exploratory purposes to understand their conceptual appropriateness within disability-related stigma measurement.

Finally, in order to minimise recall bias, given the disability context and the intersectional nature of disability-related stigma, we modified the reference period for all the scale items from 12 months in the original scales, to 3 months in our study. The questions used in our study are described in Tables 1 and 2.

**Table 1:** Enacted stigma scale items.

*For people without disabilities*				
In the past 3 months, have you witnessed or heard of a person with a disability experiencing/receiving/being (*fill in action*) from a community member because he/she has a disability?				
		Yes	No	DK^a^
Q1	Verbal abuse			
Q2	Cared for when sick			
Q3	Physical abuse/violence			
Q4	Isolated in household (made to eat alone/use separate utensils)			
Q5	Emotional support			
Q6	Gossiped about			
Q7	Financial support			
Q8	Isolated at social events			
Q9	Lost customers to buy their produce/goods or lost a job			

^a^Do Not Know.

**Table 2:** Experienced stigma scale items.

*Ask only individuals identified as having a disability*							
In the past 3 months, have you (*fill in action*) because you have a disability?					If ‘yes’, how often were you *(fill in action)* in the past 3 months?		
		Yes	No	DK^a^	Once	Sometimes	Often
Q1	Been teased, insulted or sworn at						
Q2	Been cared for when sick						
Q3	Lost housing or not able to rent housing						
Q4	Been given emotional support						
Q5	Been denied access to health care or other services						
Q6	Lost respect/standing within the community						
Q7	Been given financial support						
Q8	Been physically abused (i.e., punched, kicked, hit, etc.)						
Q9	Gained respect/standing within the community						
Q10	Been gossiped about						
Q11	Been isolated at social events						
Q12	Been discouraged from joining local groups (such as farmers groups)						
Q13	Lost customers to buy your produce/goods or lost a job						
Q14	Experienced any other positive reaction (Specify)__________________________						
Q15	Experienced any other negative reaction (Specify)________________________						

^a^Do not know.

The scale for enacted stigma collected data on negative behaviours towards people with disabilities, observed in the community in the past three months. The tool was used among farmers without disabilities and included six negative behaviour and three supportive behaviour items with the binary responses of “yes” or “no” (Table 1).

The scale for experienced stigma measured negative behaviours experienced by farmers with disabilities in the past three months and included ten negative experience and five experience of support items with the binary responses of “yes” or “no” (Table 2); and for those who answered “yes”, the frequency of these experiences: once, sometimes or often. Responses to the experienced stigma scale were recoded as follows: – where the participant responded “No” to the first question, we assigned the value “never” to the frequency question. Thus, we analysed this scale as having categorical items with 4 possible scores graded as never (0), once (1), sometimes (2) and often (3).

All tools were translated from English into three local languages – Swahili, Luo, and Luhya, and then back-translated into English. This forward and back translation of the tools, including pilot testing, were intended as a cross-cultural adaptation given the different languages used in the study catchment area as per the Beaton guidelines [26].

As the tools were previously applied in a different context and with participants with HIV/AIDS, we used factor analyses to understand, based on their correlations, how the items grouped together in our context and identify the underlying constructs they measured. We also evaluated the internal consistency of these factors [18].

### Data analysis

Statistical analyses were conducted using R version 4.4.0 [27].

Analyses were conducted separately for the enacted and experienced stigma scales on the participants without and with disabilities, respectively. First, we conducted exploratory factor analysis (EFA) to explore the underlying factor structure of each tool followed by confirmatory factor analysis (CFA) to cross-validate this structure. Finally, sensitivity analyses including measurement invariance and missing data checks were conducted.

We split the relevant analysis sample in half using the SOLOMON method [28], which creates two statistically comparable subsamples in the context of factor analyses. This strengthens cross-validation in factor analysis compared with a simple random split. EFA was conducted on the first subsample, CFA on the second. Factor analyses were based on the tetrachoric correlations for the enacted stigma questionnaire due to the dichotomous nature of the items and the polychoric correlations for the experienced stigma questionnaire due to the ordinal nature of the items [29].

#### Exploratory factor analysis.

The EFA was implemented using the R psych package version 2.3.3 [30]. Suitability of the data for factor analyses was assessed using Bartlett’s test of sphericity and the Kaiser-Meyer-Olkin (KMO) measure of sampling adequacy (MSA). KMO values above 0.7 were considered adequate and values under 0.5 unacceptable [31,32]. If the value was under 0.5, the variable with the lowest item-level MSA value was dropped iteratively till the overall KMO value reached an acceptable value [32].

We used the iterated principal factor method with an oblique rotation (oblimin), allowing for the factors to be correlated [29].

The number of factors to extract was guided by parallel analysis, Velicer MAP, inspection of Cattell’s scree plot, and theoretical considerations [29]. Where these methods gave different results, we examined the solutions sequentially from the largest to the smallest number of factors. We chose the simplest conceptually meaningful solution, in which each factor was saliently loaded by at least three items and demonstrated good internal consistency, with Cronbach’s alpha ≥0.7. Items with complex loadings were dropped. Given our sample size, a factor loading was considered as salient if ≥0.30 [32].

#### Confirmatory factor analysis.

CFA was carried out on the second subsample, using the WLSMV (weighted least square mean- and variance-adjusted) estimator as recommended for binary or ordinal data [33]. The CFA models were estimated using R package lavaan version 0.6–18 [34], and visualised using semPlot v1.1.6 [35]. We used the following recommended benchmark statistics to evaluate goodness-of-fit: RMSEA (root mean square error of approximation)<0.05, TLI (Tucker-Lewis Index)>0.95, CFI (comparative fit index)>0.95 and SRMR<0.08 [33]. The standardised CFA results are presented using standard Structural Equation Modelling (SEM) graph conventions [36], with observed variables denoted by rectangles, latent factors depicted by ovals. Unidirectional arrows represent factor loadings and curved two headed-arrows depict variances or covariances.

Reliability was estimated using McDonald’s Omega coefficient implemented by R package semTools version 0.5–6 [37]. For completeness and comparability with existing literature, Cronbach’s alpha coefficients are also reported.

#### Sensitivity analyses.

##### Measurement invariance.

We conducted measurement invariance analyses across languages on a sample subset to explore whether the measurement tools measured underlying constructs in the same way. The data on which language the tool was administered in was collected at a later stage and was therefore not available for all participants. Additionally, only two groups had sufficient sample size for further analyses: Swahili and Luo. Therefore, measurement invariance analyses were only conducted for those groups.

Given the heterogeneity between functional difficulties and psychosocial difficulties, which might affect one’s experience of stigma differently, we also conducted measurement invariance analyses for the experienced stigma scale across disability type. We used two subgroups: those who had a functional difficulty in one of the six domains but not anxiety/depression, and those who had anxiety/depression but no other functional difficulty. Individuals who had both conditions were excluded from these analyses due to small sample size.

We used multi-group confirmatory factor analysis, confirming the initial model for each group separately and then, testing models sequentially for: configural invariance (significance of the same loadings in both groups), weak invariance (constraining the factor loadings to be equal between groups), and strong invariance (additionally constraining the factor thresholds to be equal between groups). At each step, the more constrained model was retained if the chi-square test comparing the two nested models was not significant and the difference in CFI < 0.01 [38].

##### Missing data.

Among people without disabilities, the proportion of individuals with missing data to at least one question was small (1.1%). Therefore these individuals were excluded and complete-case analyses conducted on the remaining individuals. No missing data imputation was conducted.

Among people with disabilities, 7.4% had a missing response to at least one question. Due to the relatively low proportion of missingness, complete-case analyses was conducted. However, missingness was found to be associated with marital status and the presence of a physical disability (data not shown), indicating data was not missing completely at random (MCAR). We therefore carried out sensitivity analyses of the CFA, under the assumption that data were missing at random (MAR) using multiple imputation by chained equations (MICE) with 20 imputed datasets. We used R packages mice v3.17.0 [39], miceadds v3.17-44 [40] and lavaan.mi v0.1-0 [41].

### Ethics and ethical approval

The study protocol was reviewed and approved by the National Commission for Science, Technology, and Innovation (NACOSTI) (Ref #: 676151) and the institutional ethics review committee of Strathmore University (SU-IERC) (Ref #: SU-IERC1234/21). Written informed consent to participate in the study was obtained from each participant in one of three local languages (or sign language for participants with hearing impairments) via a digital consent form. All participants were paid an equivalent of US$1.5 each as a token of appreciation of their time.

## Results

Participant characteristics have been described in detail elsewhere [21].

### Enacted stigma

Among those without disability, 1.1% had missing data for at least one question and were excluded from analyses. Analyses were conducted on the remaining N = 3642 individuals. Cronbach’s alpha on the raw nine-item scale was 0.75.

#### EFA of the first subsample (N = 1821).

Bartlett’s test of sphericity was significant (p < 0.01); the KMO-MSA was 0.89, with item-specific MSA ranging from 0.78 to 0.94, indicating the data was suitable for exploratory factor analysis. Parallel analysis suggested three factors, while Velicer MAP and the scree Plot suggested two factors. These solutions were examined sequentially. The two-factor solution was retained as it met the criteria described in the Data analysis section. This solution accounted for 63% of the variance. The factor loadings from the EFA are presented in Table 3. The first factor was saliently loaded by the following items: receiving verbal abuse, experiencing physical abuse/violence, being isolated in the household, being gossiped about, being isolated at social events and losing a job or customers. We labelled this factor “negative attitudes observed towards people with disabilities”. The second factor was saliently loaded by the following items: being cared for when sick, receiving emotional support, receiving financial support. We labelled this factor “positive/supportive attitudes observed towards people with disabilities”. There was a positive correlation between the two factors (0.52).

**Table 3:** Enacted (observed) stigma scale – EFA results^a^ factor loadings.

Item	Factor 1	Factor 2
receiving verbal abuse	**0.70**	−0.03
being cared for when sick	0.16	**0.59**
experiencing physical abuse/violence	**0.82**	−0.03
being isolated in household	**0.86**	0.02
receiving emotional support	−0.05	**0.95**
being gossiped about	**0.85**	0.02
receiving financial support	0.09	**0.62**
being isolated at social events	**0.82**	0.01
lost customers to buy their produce/goods or lost a job	**0.76**	0.00

^a^Factor loadings > 0.30 indicated in bold.

#### CFA of the second subsample (N = 1821).

**Fig 1:**
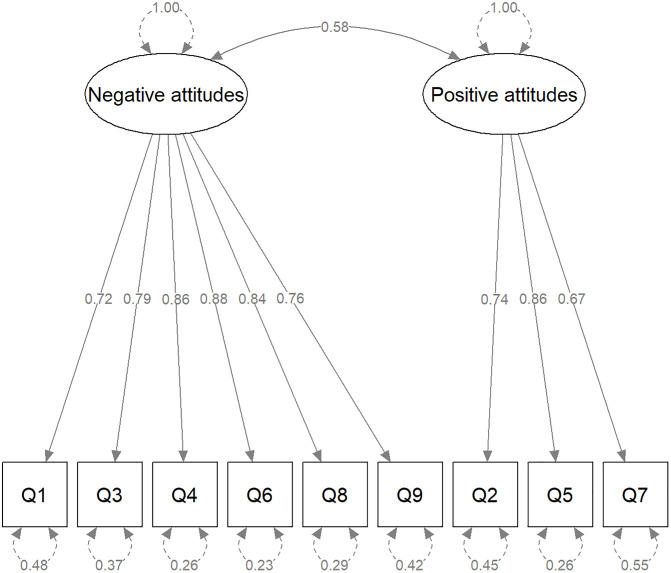
Enacted (observed) stigma in the community – CFA results.

The CFA indicated that the two-factor solution had an adequate fit to the model. All factor loadings were statistically significant, and the goodness-of-fit indices were as follows: CFI = 0.992, TLI = 0.993, RMSEA = 0.029, SRMR = 0.035. The results of the standardised CFA are illustrated in Fig 1. Internal consistency was good for the negative attitudes factor (omega = 0.79, alpha = 0.76), and was lower for the positive/supportive attitudes factor (omega = 0.66, alpha = 0.65).

#### Sensitivity analyses – Measurement invariance across language.

Data was collected using Luo for more than half of the participants without disability (55.1%), Swahili for 26.6%, English for 2.8%, Luhya, Teso or a mix of language for 1.0% and the language used was not recorded for 12.8% of participants without disability. Sensitivity analyses were conducted on the two largest groups: Swahili (N = 969) and Luo (N = 2008). We first fitted the model separately for each group, fit indices indicated adequate fit of the model in each group. For the Luo group, CFI = 0.986, TLI = 0.988, RMSEA = 0.037, SRMR = 0.042. For the Swahili group, CFI = 0.99, TLI = 0.996, RMSEA = 0.025, SRMR = 0.038. The fit indices for the configural model were: CFI = 0.989, TLI = 0.99, RMSEA = 0.034, SRMR = 0.041. Additional constraints placed for weak invariance did not significantly worsen the fit (delta chi-square = 10.3; p = 0.17, delta CFI = 0.002). The comparison of the model with weak invariance to additionally constrained the model with strong invariance also showed no significant deterioration of fit (delta chi-square = 5.4, p = 0.06, delta CFI = 0.002). These results indicated that the enacted stigma tool had configural, weak and strong measurement invariance across Luo and Swahili languages.

### Experienced stigma

Among those with disability, 7.4% had missing data for at least one question and were excluded from analyses. Analyses were conducted on the remaining N = 710 individuals. Cronbach’s alpha on the raw fifteen-item scale was 0.75.

#### EFA of the first subsample (N = 355).

Bartlett’s test of sphericity was significant (p < 0.01), indicating the variables are correlated. Initial KMO-MSA was not acceptable (0.44). Items with the lowest MSAs were iteratively dropped till reaching an overall KMO-MSA > 0.5: being cared for when sick, gained respect/standing within the community, been given emotional support, been given financial support, experienced any other positive reaction, experienced any other negative reaction, lost customers/job, being denied access to healthcare or other services, lost housing or not able to rent housing. The resulting KMO-MSA was 0.88, with item-specific MSAs ranging from 0.84 to 0.92, indicating the remaining items were suitable to factor analyses. Parallel analysis suggested two factors, while Velicer MAP and the Scree Plot suggested one factor. Solutions were examined sequentially. Only the one-factor solution met the criteria described in the Data analysis section. This solution accounted for 68% of the variance. The factor loadings from the EFA are presented in Table 4.

**Table 4:** Experienced stigma – EFA results – factor loadings^a^.

Item	Factor 1
Been gossiped about	**0.92**
Been isolated at social events	**0.86**
Been discouraged from joining local groups	**0.75**
Been teased, insulted or sworn at	**0.79**
Lost respect/standing within the community	**0.87**
Been physically abused	**0.72**

^a^Factor loadings>0.30 indicated in bold.

All remaining items loaded saliently onto the unique factor: being gossiped about, being isolated at social events, being discouraged from joining local groups such as farmer groups, being teased/insulted/sworn at, lost respect/standing within the community, being physically assaulted.

#### CFA of the second subsample (N = 355).

**Fig 2:**
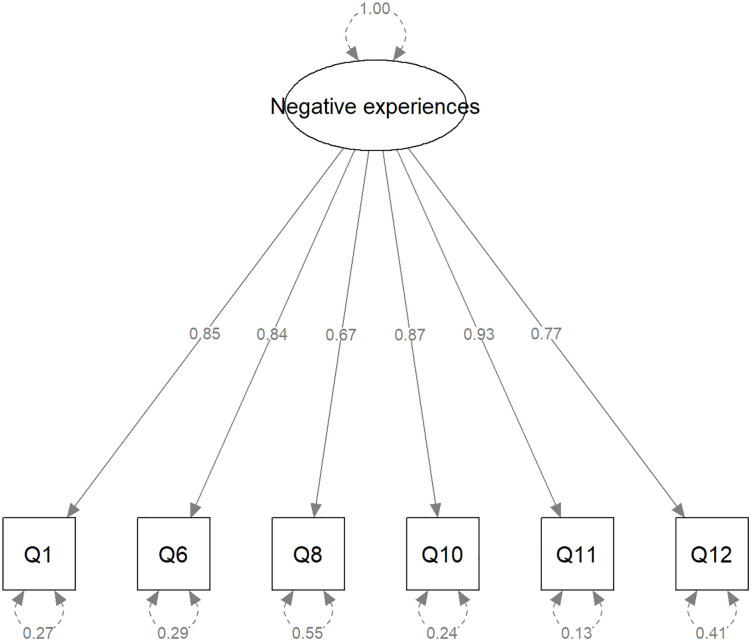
Experienced stigma – standardised results of CFA.

The CFA indicated that the one-factor solution had an adequate fit to the model, all factor loadings were statistically significant and the goodness-of-fit indices were as follows: CFI = 0.993, TLI = 0.990, RMSEA = 0.057, SRMR = 0.062. While the RMSEA was slightly higher than the benchmark, this was still acceptable, combined with the other indices meeting the benchmark criteria, indicating an adequate fit of the one-factor model. The results of the standardised CFA are illustrated in Fig 2. The factor showed good internal consistency (Omega= 0.82, alpha = 0.77).

### Sensitivity analyses

#### Measurement invariance across language.

Data was collected using Luo for most of the participants with disabilities (65.1%). Swahili was used for 20.0%, English for 1.7%, Luhya, Teso or a mix of language for only 0.4%. The language used was not recorded for 12.8% of participants with disability. Sensitivity analyses were attempted on the two largest groups: Swahili (N = 142) and Luo (N = 462). However, the model fitted in the Swahili group did not converge (probably due to small sample size). We were therefore unable to test for measurement invariance across languages for the experienced stigma tool.

#### Measurement invariance across disability type.

Among those with disabilities, 42.8% (N = 304) had a functional difficulty only (excluding anxiety or depression) 44.8% had anxiety or depression only, and 12.4% (N = 88) had both. Sensitivity analyses were conducted on the subgroups of individuals with a functional difficulty (any of the six domains) only or anxiety/depression only. Fit indices indicated adequate fit of the model when fit separately for each group. For the subgroup with any of the six domains difficulty only, CFI = 0.990, TLI = 0.986, RMSEA = 0.065, SRMR = 0.086. The RMSEA and SRMR only slightly exceeded conventional thresholds and the CFI, TLI values are well above the commonly accepted cutoffs, therefore we deemed the model to have adequate fit for this subgroup. For the anxiety/depression group, CFI = 0.990, TLI = 0.991, RMSEA = 0.056, SRMR = 0.055. The fit indices for the configural model were: CFI = 0.991, TLI = 0.988, RMSEA = 0.061, SRMR = 0.070. Additional constraints placed for weak invariance did not significantly worsen the fit (delta chi-square = 8.06; p = 0.153, delta CFI = 0.000). The comparison of the model with weak invariance to additionally constrained the model with strong invariance showed a significant deterioration of fit (delta chi-square = 38.9, p < 0.01, delta CFI = −0.05). These results indicated that the experienced stigma tool had configural and weak invariance, but not strong invariance across six-domain functional difficulty and anxiety/depression.

This indicates that factor structure and item loadings are consistent across groups, but item thresholds differed across these subgroups.

#### Results of CFA conducted on multiply imputed datasets.

Pooled results obtained from the multiply imputed datasets were very similar to the complete-case analysis results in terms of factor loadings, item variances and their significance (S5 Table). Pooled model fit indices also indicated adequate model fit: CFI = 0.963, TLI = 0.958, RMSEA = 0.02, SRMR = 0.05.

## Discussion

In this paper, we explored the factor structure and internal consistency of adapted HIV/AIDS stigma scales in the context of disability using EFA and CFA. We interpreted the findings using Stangl et al.’s Health Stigma and Discrimination (HSD) framework [42], which delineates stigma drivers and facilitators, stigma marking, and stigma manifestations across different social levels. Our adapted scales measured enacted stigma among those without disabilities and experienced stigma among those with disabilities. We identified a two-factor structure for enacted stigma: negative attitudes and behaviours (factor 1), and positive/supportive attitudes (factor 2). We identified a unidimensional factor including six items for experienced stigma. We examined measurement invariance of the enacted stigma scale across the Luo and Swahili languages. We examined measurement invariance of the experienced stigma scale across the subgroups having only a functional difficulty in one of the six domains excluding anxiety/depression and those having only anxiety/depression. Sensitivity analyses of CFA conducted on multiply imputed datasets indicated our results were robust to missing data assumptions.

We discuss below the conceptual and psychometric interpretation of our findings.

*Enacted (observed) stigma*

Factor analyses of the enacted stigma scale revealed an underlying two-factor structure. The first factor included all six negatively worded items, and showed good internal consistency. This factor reflects discriminatory attitudes and behaviours towards people with disabilities in the community, and aligns with the HSD framework’s ‘stigma practices’ within the manifestations domain (e.g., social rejection, verbal/physical aggression, exclusionary behaviour) [42].

The second factor captured positive/supportive attitudes/behaviours towards people with disabilities and included the three positively worded items about instrumental help (e.g., financial or practical support). This factor does not align with any stigma mechanism in the HSD framework. Its interpretation is unclear, particularly given its positive correlation with the negative attitudes factors. This likely reflects the complexity of human behaviours and mixed community responses. Helpful assistance can exist alongside discriminatory norms, especially in settings where structural barriers, such as limited formal social protection and exclusion from employment, leave families and communities as the main source of support [11, 43, 44]. In these settings, support can co-exist, or even arise from stigma and discrimination. For example, qualitative studies from Kenya [43], Ghana [11] and Nigeria [44] described disability as associated with a lack of capacity resulting in more dependency on others, emphasised barriers and exclusion in the workplace, and the vulnerable situation of older adults with disabilities who were often financially dependent on their children, or even had to resort to begging on the roadside. On the other hand, support can also reflect solidarity and care seen in strong familial and communal bonds across many sub-Saharan African cultures [45].

There has been debate in the literature over the fact that reverse-worded items can load onto a separate factor as a methodological artifact [46]. We do not believe the second factor to be a methodological artifact here given both positively and negatively worded items loaded positively when forced onto one factor. In addition to this, the internal consistency considerably decreased when forcing the positively worded items as reverse-coded onto one factor alongside the negative items (omega = 0.30).

Conceptually, retaining these positive/supportive items blurs stigma with support; psychometrically, they attenuated reliability. We therefore recommend dropping the positive/supportive items from disability-related stigma and discrimination measurement scales to preserve conceptual clarity.

Further research, such as qualitative studies, is needed to explore which community behaviours are genuinely experienced as supportive by people with disabilities, and enable their agency and unrestricted participation in society. This would inform the development of targeted items to measure disability-related support and understand how those align with lived experiences. Given that social support is a critical component in the wellbeing and participation in society of people with disabilities, such a measurement scale would be useful. For instance, Virendrakumar et al [47] highlighted the role of support from family, friends, communities in enabling political participation for people with disabilities.

Initial sensitivity analyses showed the scale to be measurement invariant across the Luo and Swahili languages, indicating that scale scores have the same meaning across Swahili and Luo.

Overall, the adapted scale measuring enacted (observed) stigma in the community showed promising properties.

*Experienced stigma*

The results from the experienced stigma scale were more complex. Several items were dropped, The retained unidimensional factor predominantly captured aspects of interpersonal experienced stigma including isolation, verbal stigma, loss of identity/role [19], which map onto the domain of stigma manifestations within the HSD framework.

The dropped items included all five positive/supportive experience items, all three items pertaining to loss of access to resources or livelihoods [19], and the general item pertaining to “other negative experiences”. Their removal is supported by both statistical and conceptual considerations. Their MSA was low, suggesting they were too noisy or redundant [48]. Only a small proportion of individuals experienced loss of access to resources or livelihoods (<5% for each item). The use of a shortened recall period (3 months instead of 12) may have reduced the likelihood of capturing these infrequent but important experiences. Conceptually, these items are more likely to reflect facets of structural stigma rather than everyday interpersonal stigma, it is therefore theoretically coherent that they would not load on the interpersonal stigma factor. Removing these items from our experienced stigma scale strengthens its conceptual clarity as a tool to measure “interpersonal experienced stigma” specifically.

Future iterations could explore the use of longer recall periods or more nuanced wording to better capture facets of structural stigma. For example, rather than asking about denial of healthcare, Nyblade, MacQuarrie et al. [19] asked about poorer quality health services, with further investigations on being passed from provider to provider, not given medicines etc. Hashemi, Wickenden et al. [49] showcased barriers to healthcare for people with disabilities due to attitudes and also communication, costs, logistics. These domains may warrant further exploration in relation to stigma.

The item corresponding to loss of respect or standing, which we used in our scale, correlated well with the other items. Although this item can reflect internalised stigma [20], which we did not specifically seek to measure, it can also reflect a form of interpersonal stigma, imposed by others, therefore we retained this item.

The positive/supportive items do not contribute to the measure of experienced stigma both statistically and conceptually, as detailed for the enacted scale. Therefore, we recommend dropping them from the scale. Further research is needed to better understand how people with disabilities feel supported in various contexts. Depending on study aims, simplified questions about additional care or services could be asked separately from the stigma scale (akin to Nyblade et al.’s two questions on support). Alternatively, a dedicated support scale could be developed to measure the impact of stigma-reduction interventions or help inform efforts to enhance supportive community attitudes.

Sensitivity analyses showed the scale had configural and metric invariance across those with functional difficulties only and those with anxiety/depression only. This suggests that factor structure was largely consistent across groups, although some items may be experienced differently by both subgroups. Modification indices indicated that the experience of physical abuse had lower thresholds in the anxiety/depression subgroup. This suggests that individuals with only anxiety/depression reported physical abuse at lower levels of experienced stigma than those with functional difficulties. This raises questions about whether physical abuse is a more common marker of stigma for those with anxiety/depression, a contributor to anxiety/depression, or both. This aligns with evidence that the relationship between mental health and functional difficulties can itself be complex. For example a study in South Africa [50] found disability to be associated with high depression and low self-esteem, with stigma mediating the links between these.

Another important consideration is that disability-related stigma is not limited to individuals with disabilities, it is also experienced by their caregivers or those close to them. Mothers of children with disabilities in Ghana reported they were often blamed and stigmatised for their childrens’ disability [51]. A review on epilepsy-related quality of life and stigma in Asia and Sub-Saharan Africa also highlighted high levels of stigma experienced both by people with epilepsy and those close to them [52]. Future scale development should consider expanding the experienced stigma tool to capture these experiences and inform interventions.

### Programmatic implications

Our study results imply distinct levers for intervention. First, routine monitoring of community-facing stigma reduction efforts (e.g., community dialogues, norm-change campaigns) should retain and build on the interpersonal enacted and experienced modules. Analyses and adaptation by impairment subgroup should be undertaken where feasible. Second, a short structural stigma module using a longer recall period, should be added to capture access and quality barriers in services to inform service and policy reform. Third, developing a separate disability-related support scale would help to quantify enabling, agency-enhancing support (familial, communal, programmatic) and to evaluate interventions that aim to strengthen solidarity and participation. Finally, future iterations of enacted items should move from binary to graded response options to improve sensitivity to change.

### Strengths and limitations

There is currently a dearth of validated tools to measure disability-related stigma, to allow for identifying change associated with programmatic interventions or comparisons across studies. Our large sample study has shown promising results for measuring disability-related stigma by adapting existing tools used in an HIV/AIDS context. Only a small proportion (<5%) of participants with disabilities reported other negative reactions, suggesting that there were not any significant omissions from the initial questionnaire. However, many items were dropped from the experienced stigma scale following factor analyses, resulting in a tool that mainly captured interpersonal stigma and did not include other aspects of stigma such as structural and institutional stigma. Future research is needed to validate the tool specifically as an interpersonal stigma scale or to widen its scope, and ensure content validity by involving people with disabilities and other experts in the area. We intentionally kept the tool relatively short to minimise respondent burden when used within larger studies. However, this meant we did not measure internalised stigma nor drivers of stigma which would be important to understand in stigma-reduction contexts. Additionally, cross-cultural adaptation of the tools would benefit from additional steps such as expert review, which was not conducted in this study.

The shortened recall period limits direct comparability with the original HIV stigma scales, and may have led to missing out on rarer but important stigma experiences.

Although the enacted stigma scale performed well, the use of binary yes/no items may limit the sensitivity of the scale.

Measurement invariance results were partly encouraging. While invariance was demonstrated across Luo and Swahili for the enacted scale, for the experienced stigma scale, it could not be established across languages and was only partially demonstrated across disability types. Further validation is needed to understand applicability across groups and support cross-group comparisons.

Design-based clustering could not be accounted for in these factor analyses due to software limitations. Sensitivity analyses using Stata suggested that clustering did not impact factor loading significance, although standard errors may be underestimated (see S1–S4 Tables and S1 Appendix)

Sample sizes were too small to conduct impairment-specific analyses. Future research is needed to better understand how facets of stigma might be more or less relevant for different types of impairments. Ideally, a measurement scale aiming to capture stigma across disability types should balance generalisability and relevance to specific impairments.

Disability was measured using the LFS-DM module, therefore questions on experienced stigma were asked from participants who might not self-identify as having a disability. This meant that although the study ascertained disability based on the severe degree of functional limitations, there could have been biased responses, if the fieldworkers accidentally used the word “disability” due to stigma associated with it.

Our findings might be limited in their generalisability, as our study was set in Western Kenya, an area that has been substantially affected by HIV and AIDS. The applicability of these adapted HIV scales to settings with low HIV prevalence or different disability and/or stigma contexts needs to be ascertained.

We acknowledge that there might have been social desirability bias in participants’ responses given the sensitive nature of the research topic and the high survey exposure of these settings, where participants may be more familiar with “expected” answers. Although efforts were made to mitigate for this, it is still possible that some participants may have underreported negative attitudes or overreported positive attitudes.

Finally, the experience of disability stigma does not exist in isolation and intersects with other socio-demographic factors such as poverty or gender. Women with disabilities may experience increased levels of violence [53], whereas the stigma experienced by men with disabilities may be exacerbated by patriarchal expectations around masculinity, and thus mediated by their ability to provide for their family [54]. It will therefore also be necessary to trial and validate tools of this type in diverse populations to understand how disability stigma compounds with other forms of stigma and marginalisation, and how a unified measurement tool can best capture these diverse experiences.

## Conclusion

Our study showed promising results in adapting an HIV/AIDS stigma tool to measure stigma in a broader disability context and contributed to the validity evidence of such tools. Framed within the Health Stigma and Discrimination (HSD) framework, our validated modules capture interpersonal manifestations of stigma, while structural processes require separate additional measurement scales. Future research should broaden stigma measurement to include anticipated and internalised stigma and examine subgroup differences by impairment type, with particular focus on mental health-related disabilities.

## Supporting information

S1 Table Enacted stigma – Results of CFA conducted using Stata gsem, accounting for clustering. (DOCX)

S2 Table Enacted stigma – Results of CFA conducted using Stata gsem, without accounting for clustering. (DOCX)

S3 Table Experienced stigma – Results of CFA conducted using Stata gsem, accounting for clustering. (DOCX)

S4 Table Experienced stigma – Results of CFA conducted using Stata gsem, without accounting for clustering. (DOCX)

S5 Table Experienced stigma – pooled CFA results from multiply imputed datasets. (DOCX)

S1 Appendix Design based clustering – sensitivity analyses. (DOCX)

## Abbreviations

AIDS: Acquired immunodeficiency syndrome
CFA: Confirmatory Factor analyses
DK: Don’t know
EFA: Exploratory Factor analyses
HIV: Human immunodeficiency virus
HSD: Health Stigma and Discrimination
