# Supplementary material for: Factor structure and psychometric properties of an adapted HIV stigma tool for measuring disability-related stigma among smallholder farmers in Western Kenya – Findings from a cross-sectional study
Source: PLoS One. 2026 Mar 26;21(3):e0345597. doi: 10.1371/journal.pone.0345597 (PMC13020852; doi:10.1371/journal.pone.0345597)
Supplement: S2 Table — (DOCX) [file pone.0345597.s002.docx]

Supplementary Table S2: Enacted stigma - Results of CFA conducted using Stata gsem, without accounting for clustering.

| Item | Negative attitudes factor |  |  |
| --- | --- | --- | --- |
|  | Unstandardized estimate | Robust standard error | p |
| Negative attitudes factor |  |  |  |
| Q1 | 1 | - | - |
| Q3 | 1.31 | 0.14 | <0.01 |
| Q4 | 1.62 | 0.19 | <0.01 |
| Q6 | 1.87 | 0.23 | <0.01 |
| Q8 | 1.54 | 0.18 | <0.01 |
| Q9 | 1.21 | 0.14 | <0.01 |
| Positive attitudes factor |  |  |  |
| Q2 | 1 | - | - |
| Q5 | 1.82 | 0.30 | <0.01 |
| Q7 | 0.88 | 0.10 | <0.01 |
