# Supplementary material for: Factor structure and psychometric properties of an adapted HIV stigma tool for measuring disability-related stigma among smallholder farmers in Western Kenya – Findings from a cross-sectional study
Source: PLoS One. 2026 Mar 26;21(3):e0345597. doi: 10.1371/journal.pone.0345597 (PMC13020852; doi:10.1371/journal.pone.0345597)
Supplement: S3 Table — (DOCX) [file pone.0345597.s003.docx]

Supplementary Table S3: Experienced stigma - Results of CFA conducted using Stata gsem, accounting for clustering.

| Item | Negative experiences factor |  |  |
| --- | --- | --- | --- |
|  | Unstandardized estimate | Robust standard error | p |
| Negative attitudes factor |  |  |  |
| Q1 | 1 | - | - |
| Q6 | 1.18 | 0.26 | <0.01 |
| Q8 | 0.77 | 0.28 | <0.01 |
| Q10 | 1.30 | 0.50 | <0.01 |
| Q11 | 1.58 | 0.51 | <0.01 |
| Q12 | 0.77 | 0.19 | <0.01 |
