# Supplementary material for: Factor structure and psychometric properties of an adapted HIV stigma tool for measuring disability-related stigma among smallholder farmers in Western Kenya – Findings from a cross-sectional study
Source: PLoS One. 2026 Mar 26;21(3):e0345597. doi: 10.1371/journal.pone.0345597 (PMC13020852; doi:10.1371/journal.pone.0345597)
Supplement: S5 Table — (DOCX) [file pone.0345597.s005.docx]

Supplementary Table S5: Experienced stigma – pooled CFA results from multiply imputed datasets

| Item | Negative attitudes factor |  |  |
| --- | --- | --- | --- |
|  | Standardized factor loading | p | Standardised residual variance |
| Negative attitudes factor |  |  |  |
| Q1 | 0.83 | <0.01 | 0.31 |
| Q6 | 0.84 | <0.01 | 0.29 |
| Q8 | 0.67 | <0.01 | 0.55 |
| Q10 | 0.89 | <0.01 | 0.22 |
| Q11 | 0.89 | <0.01 | 0.22 |
| Q12 | 0.73 | <0.01 | 0.47 |
