# Supplementary material for: Factor structure and psychometric properties of an adapted HIV stigma tool for measuring disability-related stigma among smallholder farmers in Western Kenya – Findings from a cross-sectional study
Source: PLoS One. 2026 Mar 26;21(3):e0345597. doi: 10.1371/journal.pone.0345597 (PMC13020852; doi:10.1371/journal.pone.0345597)
Supplement: S1 Appendix — (DOCX) [file pone.0345597.s006.docx]

**S1 Appendix – design-based clustering**

The CFA in our main results was conducted using the WLSMVS estimator from the lavaan package in R, to accommodate the ordinal nature of the items. However, to the best of our knowledge, this estimator does not currently support adjustment for clustering. To evaluate the potential impact of clustering on our CFA results, we conducted a sensitivity analysis in Stata using the gsem command, both with and without adjusting for clustering via the vce command. While this approach did not provide standardized loadings nor conventional fit indices, it still allowed us to explore the impact of accounting for clustering on the significance of the factor loadings (see S1-S4 Tables). The robust standard errors accounting for clustering were generally similar, albeit slightly higher than the unadjusted standard errors, and the significance of factor loadings remained the same. These results suggest that clustering does not markedly impact our factor analyses results, although we acknowledge that standard errors in our main results may be underestimated.
